# Expression and Characterization of 3,6-Dihydroxy-picolinic Acid Decarboxylase PicC of *Bordetella bronchiseptica* RB50

**DOI:** 10.3390/microorganisms11040854

**Published:** 2023-03-27

**Authors:** Cansheng Yuan, Lingling Zhao, Lu Tong, Lin Wang, Zhuang Ke, Ying Yang, Jian He

**Affiliations:** 1 College of Rural Revitalization, Jiangsu Open University, Nanjing 210036, China; 2 College of Life Sciences, Nanjing Agricultural University, Nanjing 210095, China; 3 Suzhou Kaisiling Environmental Sci-Technology Co., Ltd., Suzhou 215413, China

**Keywords:** picolinic acid, decarboxylase, *Bordetella bronchiseptica* RB50, biodegradation, pathogen

## Abstract

Picolinic acid (PA) is a typical mono-carboxylated pyridine derivative produced by human/animals or microorganisms which could be served as nutrients for bacteria. Most *Bordetella* strains are pathogens causing pertussis or respiratory disease in humans and/or various animals. Previous studies indicated that *Bordetella* strains harbor the PA degradation *pic* gene cluster. However, the degradation of PA by *Bordetella* strains remains unknown. In this study, a reference strain of genus *Bordetella*, *B. bronchiseptica* RB50, was investigated. The organization of *pic* gene cluster of strain RB50 was found to be similar with that of *Alcaligenes faecalis*, in which the sequence similarities of each Pic proteins are between 60% to 80% except for PicB2 (47% similarity). The 3,6-dihydroxypicolinic acid (3,6DHPA) decarboxylase gene (*BB0271*, designated as *picC_RB50_*) of strain RB50 was synthesized and over-expressed in *E. coli* BL21(DE3). The PicC_RB50_ showed 75% amino acid similarities against known PicC from *Alcaligenes faecalis*. The purified PicC_RB50_ can efficiently transform 3,6DHPA to 2,5-dihydroxypyridine. The PicC_RB50_ exhibits optimal activities at pH 7.0, 35 °C, and the *K_m_* and *k_cat_* values of PicC_RB50_ for 3,6DHPA were 20.41 ± 2.60 μM and 7.61 ± 0.53 S^−1^, respectively. The present study provided new insights into the biodegradation of PA by pathogens of *Bordetella* spp.

## 1. Introduction

Pyridine derivatives are ubiquitous in natural environments and living bodies on the earth. Pyridine derivatives, such as nicotinic acid, PLP, and NAD(P)H, play important roles in organisms that are building blocks of organs, vitamins, or cofactors of enzymatic reactions [1]. However, some pyridine derivatives produced by one organism are toxic to another one. For example, nicotine is a natural product of tobacco plant which is toxic to pests and can be used as a pesticide [2]. These pyridine derivatives compounds are widely distributed in soil, water, and sediment. Microorganisms could decompose and use them as sole carbon or nitrogen sources, and in the meantime, reduce the concentrations of those toxic compounds [1,2,3,4,5].

**Figure 1:**
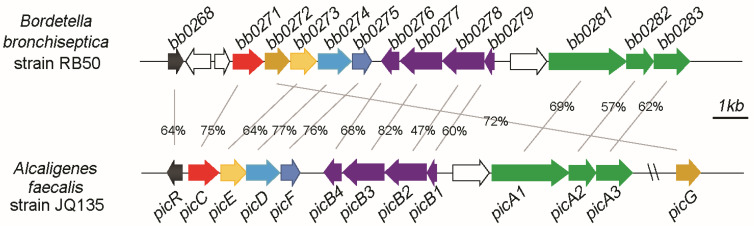
Organization of the *pic* gene clusters of *B. bronchiseptica* RB50 and *A. faecalis* JQ135. The bb0268-bb0283 indicate the gene IDs of *pic* genes in strain RB50. Numbers between the arrows indicate the percent amino acid sequence identities between strains RB50 and JQ135.

Picolinic acid (PA) is a typical mono-carboxylated pyridine derivative produced by humans/animals or microorganisms [6,7,8]. PA is an isomer of nicotinic acid. Different from nicotinic acid with a C3-carboxyl group, the carboxyl group of PA is located at the C-2 position on the pyridine ring. The N atom of PA and the O atom of carboxyl-group form a chelating structure and lead it toxic to microorganisms [9,10,11]. Several bacteria have evolved the ability to degrade PA and utilize it as sole carbon or nitrogen sources for cell growth, including Gram-positive bacteria (*Arthrobacter*, *Streptomyces*, and *Rhodococcus*) and Gram-negative bacteria (*Alcaligenes*, *Burkholderia*, and *Comamonas*) [12,13,14,15,16,17]. The PA degradation pathway and *pic* gene cluster responsible for PA catabolism have been studied in Gram-negative strain *Alcaligenes faecalis* JQ135 (Figure 1) [12,13]. The upper pathway contains intermediates 6-hydroxypicolinic acid (6HPA) and 3,6-dihydroxypicolinic acid (3,6DHPA), which are catalyzed by PA dehydrogenase (PicA) and 6HPA monooxygenase (PicB). The lower pathway was 2,5-dihydroxypyridine (2,5DHP) to fumaric acid (a Krebs cycle intermediate), which was catalyzes by four conserved enzymes, 2,5DHP 5,6-dioxygenase (PicD), N-formylmaleamic acid deformylase (PicE), maleamic acid amidohydrolase (PicF), and maleic acid isomerase (PicG). The *pic* gene cluster was predicted as widely distributed in *α*-, *β*-, and *γ*-Proteobacteria [13,18].

*Bordetella* spp. Is a large group of microorganisms, most of which cause pertussis or respiratory disease in humans and/or various animals [19,20,21]. Our previous study indicated that the *pic* gene cluster was present in strains from species of *B*. *ansorpii*, *B*. *bronchialis*, *B*. *bronchiseptica*, *B*. *flabilis*, *B*. *parapertussis*, *B*. *pertussis*, *B*. *petrii*, *B*. *pseudohinzii* and some other unclassified strains [13]. RB50 is a reference strain of genus *B. bronchiseptica* and is investigated extensively [19,21,22,23,24]. Our previous study predicted that *B. bronchiseptica* RB50 contained a putative *pic* gene cluster which similar to that of *Alcaligenes faecalis* JQ135 (13). Although the *pic* genes from strain RB50 exhibited relatively high similarity to those of strain JQ135, there was no biochemical evidence to confirm the hypothesis. 

In this study, the *picC_RB50_* gene was synthesized and its product PicC_RB50_ was over-expressed, purified, and characterized. The purified PicC_RB50_ specifically converts 3,6DHPA to 2,5DHP. The optimum conditions and the kinetic properties of PicC_RB50_ were also characterized. This study inferred that PicC_RB50_ (BB0271) has 3,6DHPA decarboxylase activity which extended our understanding of PA catabolism by the pathogen *Bordetella* spp.

## 2. Materials and Methods

### 2.1. Chemicals and Culture Media

PA, 2,5DHP, gentisic acid, and 2,3-dihydroxybenzoic acid were purchased from J&K Scientific, Ltd. (Shanghai, China). 3,6DHPA was chemically synthesized using the method as previously [12]. Methanol and formic acid for high performance liquid chromatography (HPLC) were purchased from Merck KgaA (Darmstadt, Germany). Luria-Bertani (LB) consisted of the following components (in g L^−1^): 10.0 tryptone, 5.0 yeast extract, and 10.0 NaCl. Mineral salt culture medium consists of 1.0 g (NH_4_)_2_SO_4_, 0.5 g KH_2_PO_4_·2H_2_O, 0.2 g MgSO_4_·7H_2_O, 1.5 g K_2_HPO_4_·3H_2_O, 1.0 g NaCl, at pH 7.0. The solid media were prepared by adding 20.0 g of agar powder to the above 1.0 L of liquid medium. Phosphate buffer (PBS, pH 7.4) consisted of 8.0 NaCl, 0.2 KCl, 1.42 Na_2_HPO_4_, 0.27 KH_2_PO_4_ (in g L^−1^). All other reagents used in this study were commercially available. All media were sterilized by autoclaving at 121 °C for 25 min before use.

### 2.2. Plasmids and Bacterial Strains

The plasmid pMD-19T (TaKaRa Biotech Co., Ltd., Dalian, China) was used for DNA cloning by using T-A clone method. The plasmid pET29a(+) (Novagen, New York, NY, USA) was used for the expression of the His-tagged 3,6DHPA decarboxylase PicC_RB50_ form *B. bronchiseptica* RB50. The plasmids in this study were extracted by RapidLyse Plasmid Mini Kit (Vanzyme, Nanjing, China). *Escherichia coli* DH5α was used as a host in DNA cloning experiments. *E*. *coli* BL21(DE3) strain was used as the host for over-expression of the gene cloned In the pET29a(+). *E. coli* DH5α and BL21(DE3) competent cells were purchased from TaKaRa Biotech (Dalian, China). *E*. *coli* strains were grown in LB medium under aerobic conditions at 37 °C with a rotary shaker 180 rpm.

### 2.3. Cloning the picC_RB50_ Gene

The DNA fragment containing *picC_RB50_* (963 bp, gene ID: BB0271 or AYT36_RS01365) was synthesized according to the genome of *B. bronchiseptica* RB50 [19]. The synthetic product *picC_RB50_* was ligated into plasmid pMD-19T and then sequenced (Shanghai Sangon Biotech Co., Ltd., Shanghai, China) using *E. coli* DH5α as hosts. The fragment *picC_RB50_* gene without stop coden (960 bp) was amplified using the synthesized DNA as template with primer pairs Prm-*picC_RB50_*-F 5′-ACT GCA TAT GAT GAC AAA AGT GCG CAA GAT CGC-3′ (*NdeI* restriction site underlined) and Prm-*picC_RB50_*-R 5′-ACT GCT CGA GCA GTT TGA ACA GGC GCG CGG CGT-3′ (*XhoI* restriction site underlined). The oligonucleotide primers used in this study were synthesized by a commercial company (Shanghai Sangon Biotech Co., Ltd., Shanghai, China). PCR was performed in a programmable thermocycler. The PCR procedures were an initial denaturing step of 5 min at 95 °C, 30 cycles of 95 °C for 15 s, annealing step at 56 °C for 20 s, elongation step at 72 °C for 1 min and a final elongation step at 72 °C for 5 min. The PCR mixture contained 0.2 U/μL of EX Taq polymerase (TaKaRa, Dalian, China), 0.25 mM concentrations of each deoxynucleoside triphosphate, 0.5 μM concentrations of each primer, 1× buffer, ∼50 ng of DNA, and water to 50 μL. The PCR products were determined by standard gel electrophoresis and purified by the PCR product purification kit (Vanzyme, Nanjing, China). The amplified fragment *picC_RB50_* gene was digested with QuickCut restriction enzymes with a 30 μL reactioin mixture of 10 mM Tris-HCl (pH7.5), 100 mM KCl, 0.1 mM EDTA, 1 mM DTT, 0.01% BSA, 0.15% TritonX-100, 50% Glycerol, 1 μg of DNA, 1 μL QuickCut *NdeI* and 1 μL QuickCut *XhoI*. The plasmid pET29a(+) were digested with *NdeI* and *XhoI* with the same procedures. Then, the above DNA fragements and plasmids were ligated together with the One Step Cloning kit (Clontech, Beijing, China) according to the manufacturer’s instructions. The rsesulting plasmid pET-*picC_RB50_* were then transferred into *E*. *coli* BL21(DE3) strain for overexpression of PicC_RB50_ which contained C-terminally 6× His-tag. The final recombinant plasmid was verified by DNA sequence. 

### 2.4. Expression and Purification PicC_RB50_

The recombinant plasmid pET-*picC_RB50_* was transformed into *E*. *coli* BL21(DE3) for protein overexpression. The BL21(DE3) cell was grown at 37 °C to an optical density OD_600_ of 0.4 in LB supplemented with 50 mg/L kanamycin. C-terminally His-tagged PicC_RB50_ expression was induced by the addition of 0.1 mM isopropyl β-D-1stiogalactopyranoside (IPTG) at 16 °C for 12 h when the cell optical density at 600 nm reached to 0.35. The cells were harvested by centrifugation at 12,000 rpm for 5 min, draw away the supernatant, resuspended and washed twice with 50 mM PBS (pH 7.4) buffer. The induced BL21(DE3) cells were re-suspended in the combination buffer (40 mM Tris-Cl, 0.5 M NaCl, and 5 mM imidazole, pH 8.0) and disrupted by sonication (Ultrasonic Cell Crusher XO-900D) in an ice-water bath for 15 min (on for 2 s and off for 3 s). The cell-free extract was removed by centrifugation at 12,000 rpm for 30 min at 4 °C. The supernatants were loaded onto an Ni-NTA (Shanghai Sangon Biotech Co., Ltd., Shanghai, China) column, which was pre-equilibrated with combination buffer with 20 mM imidazole (pH 8.0). The column was eluted with 100 mL, 50 mL, and 10 mL combination buffer of 50 mM, 100 mM, and 300 mM imidazole (pH 8.0), respectively. Purified recombinant PicC_RB50_ should be dialyzed against buffer to remove imidazole with a Spectra/Por CE dialysis membrane with a molecular weight cutoff of 3500 (Spectrum Laboratories, Inc., Shanghai, China) at 4 °C for 12 h. The purified PicC_RB50_ was monitored by 12% sodium dodecyl sulfate-polyacrylamide gel electrophoresis (SDS-PAGE). In addition, two site-directed mutagenesis of PicC_RB50_ were constructed and over-expressed, in which histidine residues were changed into alanines, i.e., PicC(H163A) and PicC(H216A). For example, to construct PicC(H163A), the mutant *picC_RB50_* fragments were amplified using plasmid pET-PicC_RB50_ as DNA templates through overlap PCR. Two primer pairs Prm-*picC_RB50_*-F and Mut-H163A-1 (5′-GAC GTG CCG CTG TAC CTG GCG CCG TTC GAC GCC TAC GTG-3′) (alanine encoding codon underlined) and primer pairs Mut-H163A-2 (5′-CAC GTA GGC GTC GAA CGG CGC CAG GTA CAG CGG CAC GTC-3′) (alanine encoding codon underlined) and Prm-*picC_RB50_*-R were used to amplify two fragments of *picC_RB50_* gene with the coden of His 163 (CAT) to Ala (GCG). The PCR procedures were set as an initial denaturing step of 5 min at 95 °C, 30 cycles of 95 °C for 15 s, annealing step at 56 °C for 20 s, elongation step at 72 °C for 20 s and a final elongation step at 72 °C for 2 min. After that, two PCR products were purified by the PCR product purification kit (Vanzyme, Nanjing, China) and then used as DNA templates for the second PCR. In the second PCR procedure, there only 10 amplification cycles 95 °C for 15 s, annealing step at 56 °C for 20 s, elongation step at 72 °C for 1 min. Additionally, then Prm-*picC_RB50_*-F and Prm-*picC_RB50_*-R were added for the third round PCR with procedures of an initial denaturing step of 5 min at 95 °C, 30 cycles of 95 °C for 15 s, annealing step at 56 °C for 20 s, elongation step at 72 °C for 20 s and a final elongation step at 72 °C for 5 min. The final PCR products were purified by the PCR product purification kit and then were ligated into *NdeI* and *XhoI*-digested plasmid pET29a(+) via the One Step Cloning kit (Clontech, Beijing, China) according to the manufacturer’s instructions. The reuslting plasmid pET-PicC(H163A) were transferred into *E*. *coli* BL21(DE3) strain for overexpression of protein PicC(H163A). Resultant constructs were confirmed by DNA sequencing. The expression and purification of the mutant PicC proteins were performed as described in the section above. 

### 2.5. Enzyme Assay of Purified His-Tagged PicC_RB50_

For the 3,6DHPA decarboxylase activity, the enzyme reaction mixture contained 50 mM PBS (pH 7.4), 0.2 mM 3,6DHPA, and 10 μg of purified PicC_RB50_ (in 1 mL) and was incubated at 25 °C. Total protein concentrations were determined using the Bradford method [25]. Conversion of the 3,6DHPA by PicC_RB50_ was continuously determined by UV/Vis spectrophotometry (Shimadzu, UV-2450, Japan) at 25 °C. The enzymatic activities were calculated spectrophotometrically by the disappearance of 3,6DHPA at 360 nm (ε = 4.4 cm^−1^ mM^−1^) according to previous studies [12]. The product 2,5DHP was confirmed via LC-MS/MS analysis as described below. The optimum pH of PicC_RB50_ was determined using the following buffers: 50 mM citric acid-sodium citrate (pH 4.0 to 6.0), 50 mM KH_2_PO_4_-K_2_HPO_4_ (pH 6.0 to 8.0), and 50 mM glycine-NaOH (pH 8.0 to 9.8) at 25 °C. The optimum temperature of the PicC_RB50_ was determined to be between 5 and 60 °C in PBS (pH 7.4). The influences of heavy metals were performed by adding 1 mM Ag^+^, Ca^2+^, Co^2+^, Cu^2+^, Hg^+^, Fe^3+^, Mg^2+^, Mn^2+^, Ni^2+^, Zn^2+^, and the metal ion chelating agent EDTA, respectively. Purified PicC_RB50_ was pre-incubated with various metal ions and inhibitors at 4 °C for 30 min to study their effects on the enzyme. The activity was expressed as a percentage of the activity obtained in the absence of the added compounds. To determine the effect of one condition, other conditions were kept at fixed concentration of the standard reaction mixture and the reaction was started by the addition of 3,6DHPA. To determine the kinetic constants for 3,6DHPA, a range of 3,6DHPA concentrations (0 to 300 μM) was used. The values were calculated through nonlinear regression fitting to the Michaelis–Menten equation. One unit of the activity was defined as the amount of enzyme that catalyzed 1 μmol of 3,6DHPA in 1 min at pH 7.0 and 25 °C. 

### 2.6. Analytical Methods

The degradation of the substrates by PicC_RB50_ was analyzed by High Performance Liquid Chromatography (HPLC) with a C18 reversed phase column (5 μm, 4.60 mm × 250 mm) (Thermo Fisher Scientific, Waltham, MA, USA). The concentrations of the compounds were calculated using standard samples. The mobile phase consisted of methanol, water, and formic acid (12.5:87.5:0.2 [*v*/*v*/*v*]) at a flow rate of 0.8 mL/min at 30 °C. The UV-VIS spectra (260 nm to 400 nm) were observed by an Evolution 201 spectrophotometer (Thermo Fisher Scientific, Inc., Waltham, MA, USA). LC/TOF-MS analysis was performed in a TripleTOF 5600 (AB SCIEX) mass spectrometer as described previously [26].

## 3. Results

### 3.1. Pic Gene Cluster Present in Bordetella *spp*.

**Figure 2:**
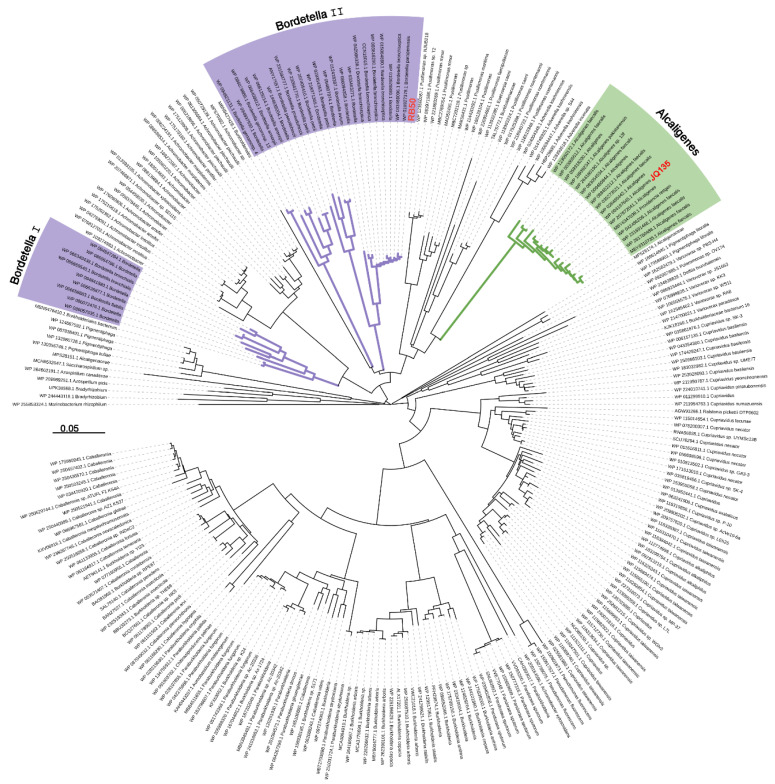
Phylogenetic relationships of PicC from strains RB50, JQ135, and other related bacteria. The amino acid sequences of PicC_RB50_ of *B. bronchiseptica* strain RB50 was selected for comparisons against the non-redundant protein sequences database in NCBI using Blastp (protein–protein BLAST). The expected (E) value inclusion threshold was 10. Strains containing PicC_RB50_ homologues with coverage >60% and identity >60% were collected and further assessed for the presence of PicA, PicC, and PicGEDF. Only bacteria containing all pic genes were selected and PicC were used for phylogenetic tree construction. Accession numbers of sequences are given before genus names. Bar, 0.05 substitutions per amino acid residue position. The PicC sequences from *Bordetella* and *Alcaligenes* strains were indicated in purple and green, respectively. PicC form strain RB50 (WP_003807348.1) and strain JQ135 (WP_094197645.1) were in red.

*Bordetella* spp. strains can cause pertussis or respiratory disease in humans and/or various animals [19,20]. *B*. *bronchiseptica*, *B*. *parapertussis*, and *B*. *pertussis* are three classical *Bordetella* species which are highly concerned due to their pathogenicity and could invade the respiratory tract of animals or human and cause severe diseases. *B*. *petrii* is the only environmental species with remarkable abilities to degrade aromatic compounds isolated from polluted soil, river sediment, or grass root [27]. To date, there are 25, 90, 637, and 1 complete genome sequences released in NCBI genome database in *B. bronchiseptica*, *B*. *parapertussis*, *B*. *pertussis*, and *B. petrii* strains, respectively. After genomic survey, all these *Bordetella* strains in these four species contains the *pic* gene cluster (Figure 1 and Figure 2). The protein sequences, taking PicC as an example, of PicC homologues from *Bordetella* spp. showed 60–70% identities against that of PicC. Phylogenetic tree showed that the PicC of most *Bordetella* bacteria were clustered into two branches and separated from other species such as *Alcaligenes* species (Figure 2).

### 3.2. Organization of Pic Gene Cluster of B. bronchiseptica RB50

*B. bronchiseptica* strain RB50 is a representative model of *Bordetella* species which has been studied in depth [19,23,24]. The organizations of the *pic* gene cluster of strain RB50 were found to be similar to that of *A. faecalis* JQ135 (Figure 1). For example, *picT* and *picB* form a divergent group. *picA1A2A3* and *picB1B2B3B4* were clustered together similarly. Only a few individual genes are organized differently. For example, in *A. faecalis* JQ135, the *picG* gene is the distance from *pic* cluster, while in *B. bronchiseptica* RB50, the *picG* gene is located between *picC* and *picE*. The *picR* gene was separated with two unknown genes from *picC* gene in *B. bronchiseptica* RB50. The *picGEDF* encodes 2,5DHP 5,6-dioxygenase, N-formylmaleamic acid deformylase, maleamic acid amidohydrolase, and maleic acid isomerase, respectively, which catalyze 2,5DHP to fumarate (an intermediate of TCA). The organization of *picGEDF* of *B. bronchiseptica* RB50 is consistent with most other 2,5-DHP degradation gene clusters, e.g., *hpo* cluster from *P. putida* S16 [28]. The protein sequence similarities of each gene are between 60% and 80% with an exception of PicB2 (47% similarity).

### 3.3. Cloning and Over-Expression of PicC_RB50_

**Figure 3:**
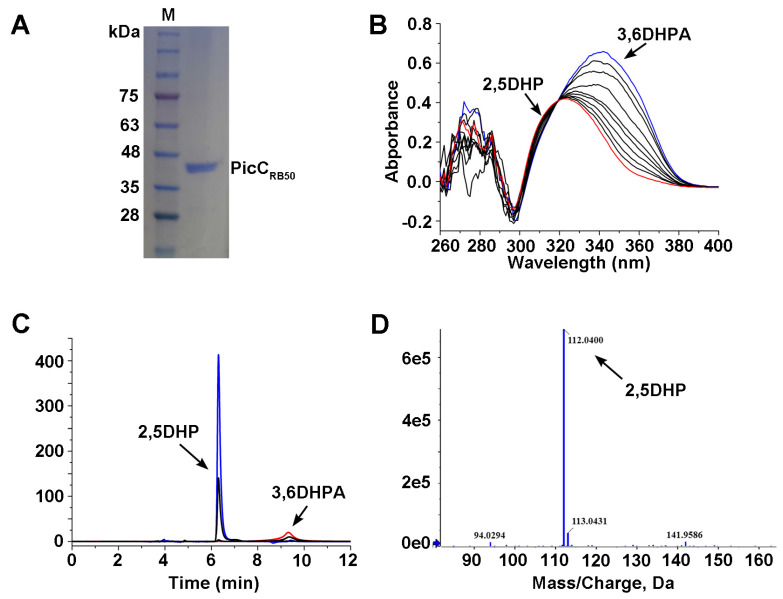
Purification and identification of PicC_RB50_. (**A**) SDS−PAGE analysis of purified PicC_RB50_. Lane M, protein marker. A single bond of purified PicC_RB50_ was indicated. (**B**) Spectrophotometric monitor of 3,6DHPA consumption (blue) and 2,5DHP formation (red) by purified PicC_RB50_. (**C**) HPLC profiles of 3,6DHPA consumption and 2,5DHP formation by purified PicC_RB50_. (**D**) LC/TOF−MS profile of the transformation product 2,5DHP.

The homologous protein of PicC from *B. bronchiseptica* RB50 (BB0271, WP_003807348.1, designated as PicC_RB50_) was annotated as amidohydrolase family protein or Amidohydro-rel domain-containing protein in public database (e.g., NCBI). In order to assess the hypothesis, the DNA sequence of *picC_RB50_* gene was synthesized and ligated in plasmid pET29a yielding pET-PicC_RB50_. The synthesized *picC_RB50_* gene product and the recombinant plasmid pET-PicC_RB50_ were checked by agarose gel electrophoresis. Then, the pET-PicC_RB50_ was transferred into *E. coli* BL21(DE3) and the recombinant 6× His PicC_RB50_ was over-expressed and purified (Figure 3A). The molecular mass of PicC_RB50_ was approximately 40.2 kDa and consisted of a single polypeptide as observed by SDS-PAGE. 

### 3.4. The PicC_RB50_ Catalyzes 3,6DHPA into 2.5DHP

The enzymatic activity of purified PicC_RB50_ towards 3,6DHPA was first monitored spectrophotometrically with a UV-VIS absorption at 260 nm to 400 nm. As shown in Figure 3B, the UV-VIS absorption curves were changed significantly. The 3,6DHPA consumed with a decrease at maximum 340 nm. As a result, a product accumulated at 250 nm which accord to absorption spectra of 2,5DHP. HPLC results indicated that 3,6DHPA (retention time 9.3 min) was degraded and transformed to a product, which had the same retention time as authentic sample of 2,5DHP (6.4 min) (Figure 3C). LC/TOF-MS analysis showed that the molecular ion peak of the product was 112.0400 (M + H^+^) which was consistent with standard 2,5DHP (Figure 3D). PicC_RB50_ showed no activities to gentisic acid and 2,3-dihydroxybenzoic acid which were structural analogues of 3,6DHPA, indicating it specific for 3,6DHPA. In addition, the functions of key histidine residues of PicC_RB50_ were determined by site-directed mutagenesis. The resultant mutant proteins PicC(H163A) and PicC(H216A) lost the abilities of 3,6DHPA decarboxylase activity completely, indicating these two histidine residues were crucial for PicC_RB50_.

### 3.5. Characteristics of the PicC_RB50_

**Figure 4:**
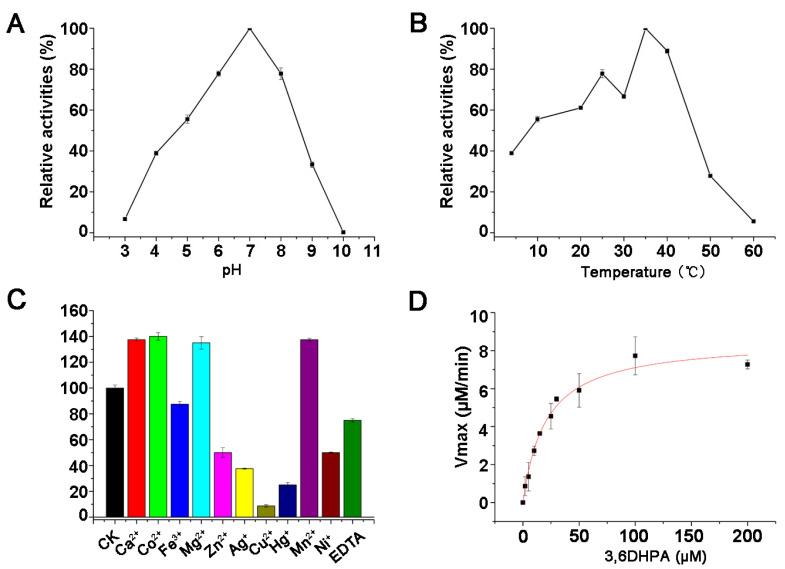
Characterization of PicC_RB50_. (**A**) Effect of pH on enzyme activity of PicC_RB50_. (**B**) Effect of temperature on enzyme activity of PicC_RB50_. (**C**) Effect of metal ions on enzyme activity of PicC_RB50_. (**D**) The kinetic curve of PicC_RB50_. The Data were shown in means ± S.E.M.

The optimum pH for PicC_RB50_ activity was determined at pH 7.0. It retained less than 10% relative activity at pH 3.0 or 10.0 (Figure 4A). The optimum temperature for the PicC_RB50_ activity was determined to be 35 °C, and PicC_RB50_ show relatively high activity at temperatures from 20 °C to 40 °C (Figure 4B). PicC_RB50_ was relatively stable at 35 °C and remains approximately 80% and 40% activity after 2 h and 12 h, respectively. When temperatures were above 40 °C, PicC_RB50_ lost its stability easily. PicC_RB50_ was strongly inhibited by 1 mM of the heavy metals Ag^+^, Cu^2+^, and Hg^+^, whereas it was slightly inhibited by Fe^3+^, Ni^2+^, Zn^2+^, and the metal ion chelating agent EDTA. In contrast, Ca^2+^, Co^2+^, Mg^2+^, and Mn^2+^ significantly increased PicC_RB50_ activity (Figure 4C). The enzyme kinetics of the recombinant PicC_RB50_ showed that *K_m_* and *k_cat_* values for 3,6DHPA were 20.41 + 2.60 μM and 7.61 ± 0.53 S^−1^, respectively (Figure 4D). 

## 4. Discussion

In this study, the function of 3,6DHPA decarboxylase PicC_RB50_ of *B. bronchiseptica* RB50 was investigated in vitro which can transform 3,6DHPA to 2,5DHP. The PicC_RB50_ exhibited optimal activities at pH 7.0, 35 °C, and the *K_m_* and *k_cat_* values of PicC_RB50_ for 3,6DHPA were 20.41 + 2.60 μM and 7.61 ± 0.53 S^−1^, which were similar with those of PicC_JQ135_ from *A. faecalis* JQ135 [12]. The study proved our previous prediction that *B. bronchiseptica* RB50 contained a *pic* gene cluster and had the potential to degrade PA [13]. 

PicC_RB50_ was annotated as amidohydrolase family protein or Amidohydro-rel domain-containing protein in *B. bronchiseptica* genome. Previous studies found that *picC_RB50_* gene (BB0271) was adjacent to a conserved cluster whose products catalyze 2,5DHP to fumarate [24]. However, no further investigations or predictions were carried out at that stage. Our present study proved that BB0271 is actually 3,6DHPA decarboxylase. In addition, strain *B. bronchiseptica* RB50 was reported to be capable of degrading nicotinic acid, in which 6-hydroxy-nicotinic acid and 2,5DHP were key intermediates. In *B. bronchiseptica* RB50, there were two identical copies of *picGEDF* genes (≈100% identical in DNA sequences). One was located in *pic* gene cluster involved in PA degradation based on the present study. The other was located in *nic* gene cluster involved in nicotinic acid degradation [24]. Rare reports showed two identical gene clusters existing in one bacterium for different pyridine derivatives [13]. The possible reason might be due to a shortcut combination of horizontal gene transfer of *picGEDF* genes and the same intermediate 2,5DHP. 

PA is a natural pyridine-derivative which can be produced both by human/animals and microorganisms [6,7,8]. As reported by previous studies, *B. bronchiseptica* RB50 and most other *Bordetella* spp. strains are pathogens of humans, birds, and animal livestock [19,21]. A question raised here is whether *Bordetella* strains degrade PA when parasitizing the host. In human body, PA is a metabolite of L-tryptophan metabolite and present in various biological issues, such as cell culture supernatants, serum, and human milk [7]. Rare reports have shown PA could be deposed by human. PA was reported as a neuroprotectant and plays a role in the pathogenesis of inflammatory disorders within the central nervous system [29]. In addition, PA is toxic to bacteria. For examples, PA was reported to reduce the degradation efficiency of nitrobenzene and could inhibit sporulation of *Bacillus cereus* [30]. Thus, if *Bordetella* strains degrade PA and utilized it as carbon source, the subsequent results could be (i) removing and decomposing the inhibitor on pathogens; (ii) utilizing the inhibitors as nutrients for cell growth; and (iii) enhancing the pathogenicity of *Bordetella* strains. Therefore, it will be an interesting topic to check whether PA exists in the diseased tissue and can be decomposed by *Bordetella* strains in vivo.

PA is also produced by microorganisms during degradation of some aromatic compounds. For example, PA was a byproduct of diquator or nitrobenzene [6,8]. Thus, PA comes to nutrients for soil/water bacteria. Bacteria from species *B*. *petrii* are isolated from environmental niches, such as polluted soil, river sediment, or grass root [27]. Bioinformatic analyses also showed that *B*. *petrii* harbors the *pic* cluster and the PicC was 84% sequence similarities to PicC_RB50_. Therefore, we can infer that PA can be degraded and served as nutrients for *B*. *petrii* strains. Bacteria in nature often face complex and nutrient-limiting environments [31,32]. Environmental *Bordetella* strains with *pic* cluster are bound to survive when facing the PA-containing micro-environments.

## Data Availability

The 963-bp *picC_RB50_* gene is avalaible on GenBank under accesion no. of BB0271 or AYT36_RS01365.
